# Effects of Lower Limb Position and Peri-Hip Muscle Co-Contraction on the Efficiency of Voluntary Pelvic Floor Muscle (PFM) Contraction During PFM Exercises

**DOI:** 10.7759/cureus.83088

**Published:** 2025-04-27

**Authors:** Yoshinobu Sato, Yuki Miyake, Masaaki Nakajima

**Affiliations:** 1 Physical Therapy, Studio TAIKA, Fukuyama, JPN; 2 Occupational Therapy, School of Health Science and Social Welfare, Kibi International University, Takahashi, JPN; 3 Physical Therapy, School of Health Science and Social Welfare, Kibi International University, Takahashi, JPN

**Keywords:** dual task training, healthy adult, pelvic floor muscle, ultrasound image, urinary

## Abstract

Pelvic floor muscle (PFM) exercises are essential for managing stress urinary incontinence (SUI), and various approaches, including differing lower limb positions and the use of assistive devices, have been proposed to enhance their effectiveness. This study aimed to evaluate how different lower limb positions and co-contraction of peri-hip muscles affect the efficiency of voluntary PFM contractions. Nineteen healthy female participants performed four types of PFM exercises: supine (Exercise A, Ex-A), supine kneeling (Exercise B,Ex-B), supine kneeling with hip abduction (Exercise C,Ex-C), and supine kneeling with hip adduction (Exercise D,Ex-D). Bladder base elevation, as an indicator of PFM contraction efficiency, was assessed using ultrasound. The results showed that the supine kneeling position (Ex-B) produced significantly greater bladder base elevation compared to the standard supine position (Ex-A) (p < 0.01). However, adding hip abduction or adduction (Ex-C and Ex-D) led to significantly lower elevations than Ex-B (p < 0.01), suggesting that co-contraction of the peri-hip muscles may reduce the efficiency of voluntary PFM contractions. This study indicates that the supine kneeling position is the most effective posture for PFM exercises, emphasizing the importance of avoiding unnecessary peri-hip muscle engagement to maximize contraction efficiency. These findings may help improve exercise protocols for preventing and managing SUI. However, a limitation of the study is the exclusive inclusion of young, healthy women; further research is needed in SUI patients or at-risk populations to confirm these results and develop more targeted exercise strategies.

## Introduction

Urinary incontinence (UI) affects approximately 30-50% of adult women [1-3]. Among the various types of UI, including functional and urge incontinence, stress UI (SUI) is the most common, accounting for 40-50% of cases, and is primarily caused by pelvic floor muscle (PFM) dysfunction [2,4,5]. SUI is also closely linked to reduced quality of life [4,6,7].

PFM exercises are recognized as an effective intervention for preventing stress-related UI [8-10] and are recommended as a Grade A treatment in the Japanese guidelines. However, about 30% of patients struggle to voluntarily contract their PFMs [11-14].

Voluntary contraction of the PFMs is believed to occur in coordination with other muscle groups, including the hip adductors, abductors, and core muscles [15,16]. Some experts recommend performing PFM exercises alongside contraction of the hip adductors or abductors. However, studies have shown that simultaneous contraction with these muscle groups may not enhance the efficiency of PFM activation [17]. Additionally, there is no consensus on the optimal exercise posture, such as whether the exercises should be performed in the supine position [18] or in a kneeling position [19].

This study, therefore, aimed to investigate which lower limb position yields the most efficient voluntary PFM contraction and to explore the impact of co-contraction of the peri-hip muscles during PFM exercises.

## Materials and methods

Experimental protocol

Nineteen healthy female university students (mean age: 20.4 ± 1.1 years; BMI: 20.1 ± 1.9 kg/m²) participated in this study. Inclusion criteria were as follows: (1) no history of orthopedic or neurological conditions affecting the trunk or hip joints; (2) no complaints of lower back pain, except during menstruation; and (3) no history of childbirth or pelvic floor disorders. All participants provided informed consent prior to participation.

Participants were instructed to refrain from eating for two hours before the experiment. One hour before testing, they were asked to void their bladder and then consume 500 ml of water immediately prior to the start of the experiment [11].

Participants lay in the supine position on a treatment bed and performed four randomly assigned exercise tasks (Exercise A (Ex-A), Exercise B (Ex-B), Exercise C (Ex-C), and Exercise D (Ex-D)). Pelvic floor elevation during each task was evaluated using ultrasound imaging.

Exercise tasks

Four types of PFM exercise tasks were performed. Ex-A involved voluntary PFM contraction in the supine position with hips in neutral (0° flexion). Ex-B involved contraction in the supine kneeling position with the hips flexed at 45°. Ex-C was the same as Ex-B but included isometric contraction of the hip abductor muscles, while Ex-D combined Ex-B with isometric contraction of the hip adductor muscles. In each task, participants performed voluntary PFM contractions for 10 seconds [19], followed by a 20-second rest interval. Each task was performed three times. The verbal cue for contraction was standardized as “Please tighten your buttocks as if you were pulling your vagina up inside” [20]. Participants were instructed to breathe normally throughout the exercises [20].

PFM Contraction in Supine Position (Ex-A)

**Figure 1:**
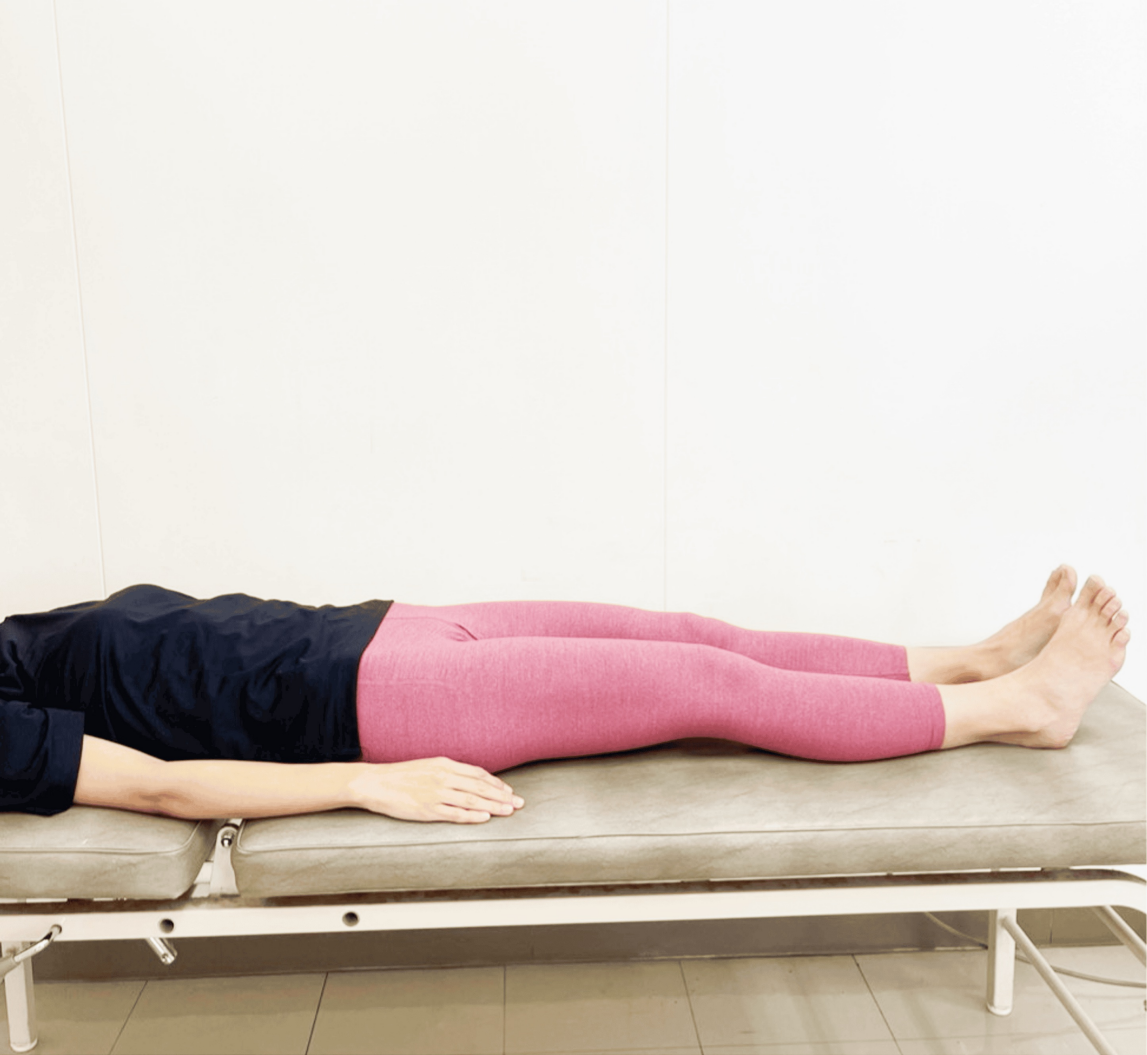
PFM contraction in the supine position (Ex-A) Ex-A, Exercise A; PFM, pelvic floor muscle

The participant was positioned in a supine position on the treatment table and performed PFM contractions (Figure 1).

PFM Contraction in the Supine Kneeling Position (Ex-B)

**Figure 2:**
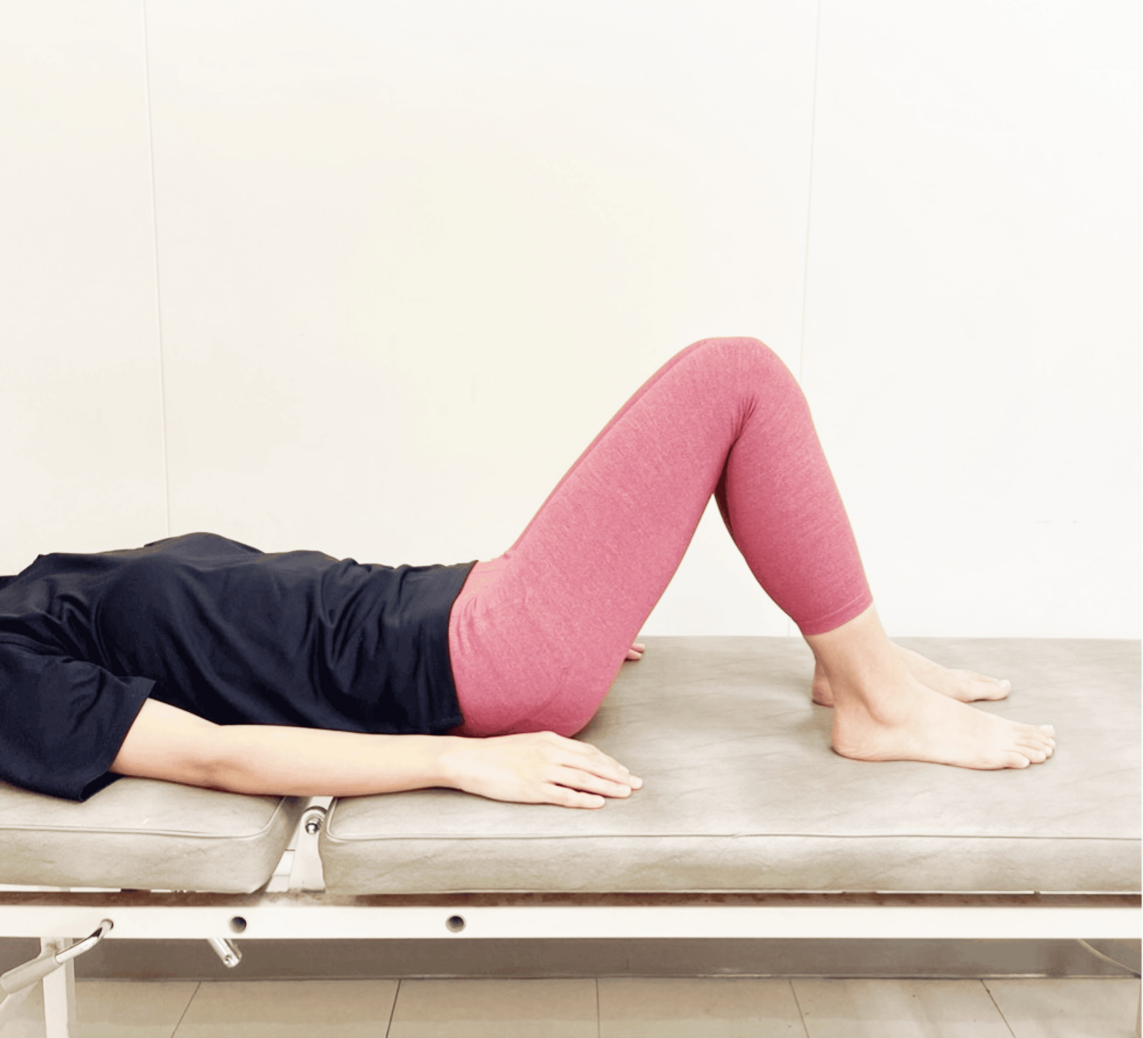
PFM contraction in the supine kneeling position (Ex-B) Ex-B, Exercise B; PFM, pelvic floor muscle

The participant was positioned on the treatment table in a supine kneeling position with 45° hip flexion and neutral hip rotation, and PFM contractions were performed (Figure 2).

PFM Contraction While Performing Abduction in the Supine Kneeling Position (Ex-C)

**Figure 3:**
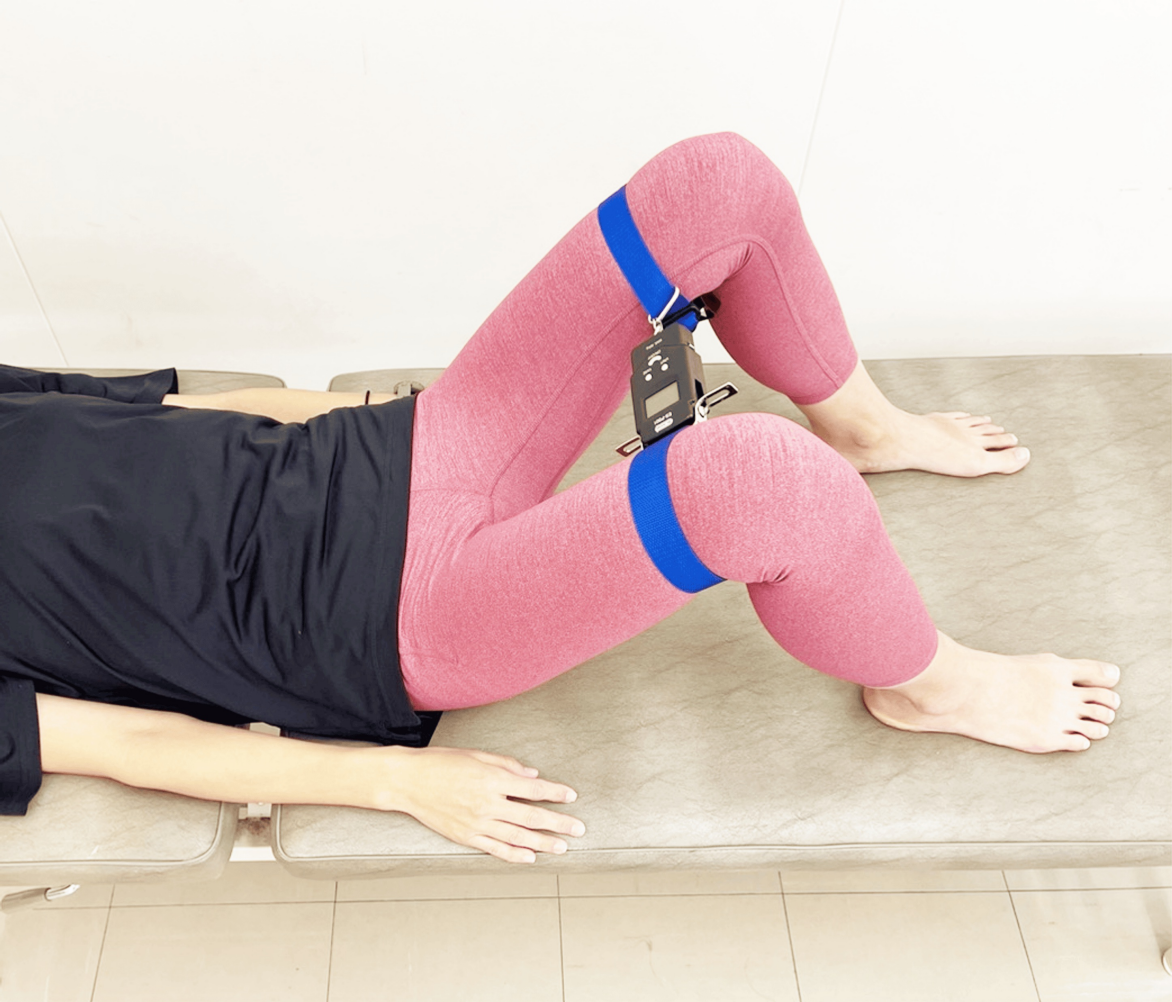
PFM contraction while performing abduction in the supine kneeling position (Ex-C) Ex-C, Exercise C; PFM, pelvic floor muscle

The participant was positioned in a supine kneeling position (hip flexion: 45°; internal/external rotation: 0°) on the treatment table, and a band with a pull sensor was attached to both thighs (approximately 5 cm proximal to the lateral epicondyle) to facilitate abduction and external rotation of the hip joints. The participant then performed PFM contractions while maintaining this position with abduction and external rotation of the hips (Figure 3). The abduction force was adjusted to 30% of the value measured during maximal voluntary abduction effort [17].

PFM Contraction While Performing Adduction in the Supine Kneeling Position (Ex-D)

**Figure 4:**
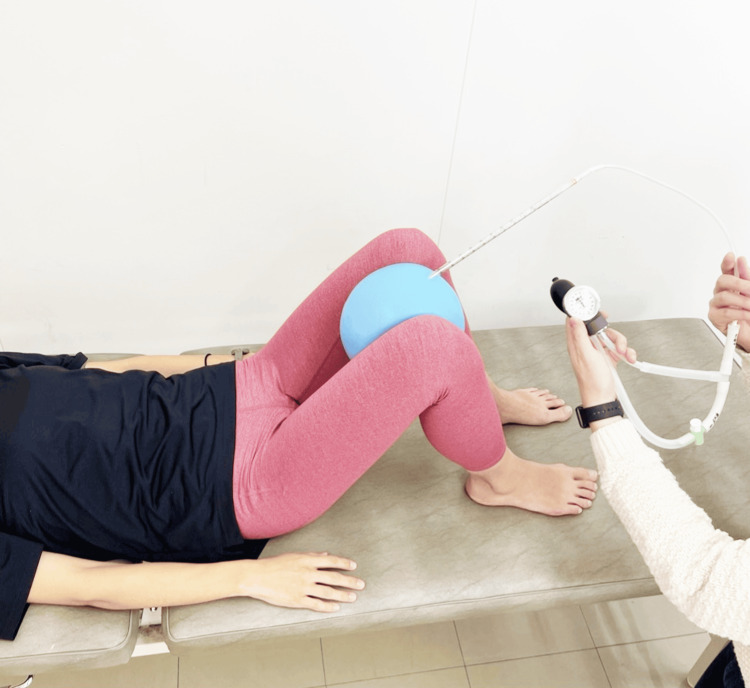
PFM contraction while performing adduction in the supine kneeling position (Ex-D) Ex-D, Exercise D; PFM, pelvic floor muscle

The participant was positioned in a supine kneeling position (hip flexion: 45°; abduction: 30°) on a treatment table, and a ball equipped with a pressure gauge was placed between the thighs (5 cm proximal to the medial epicondyle) to facilitate PFM contraction (Figure 4). The participant then performed PFM contractions while simultaneously adducting the hip joints. The adduction force was adjusted to 30% of the value measured during maximal voluntary adduction effort [17].

Evaluation of the degree of the PFM voluntary contraction

**Figure 5:**
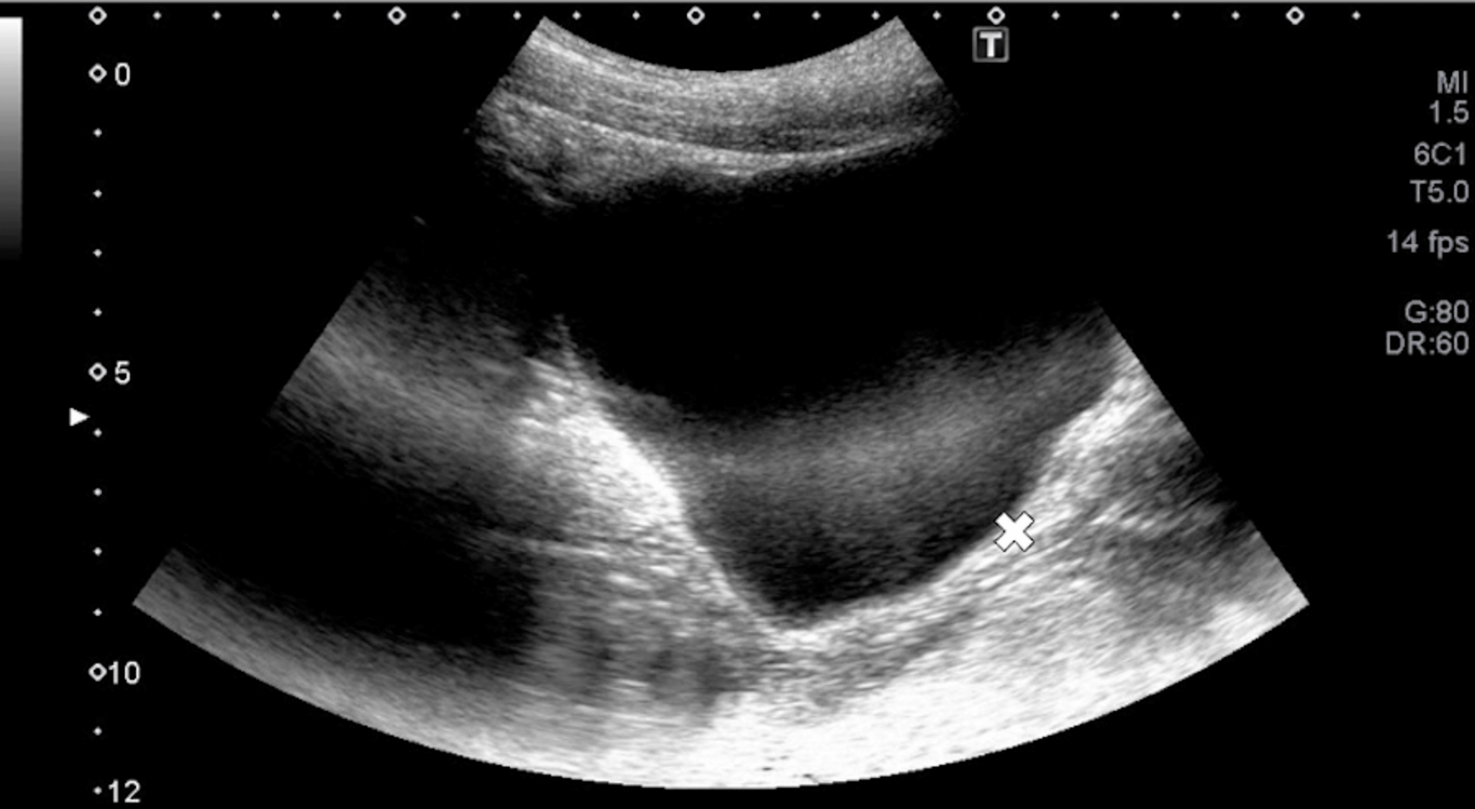
Ultrasound image of bladder base elevation at rest

**Figure 6:**
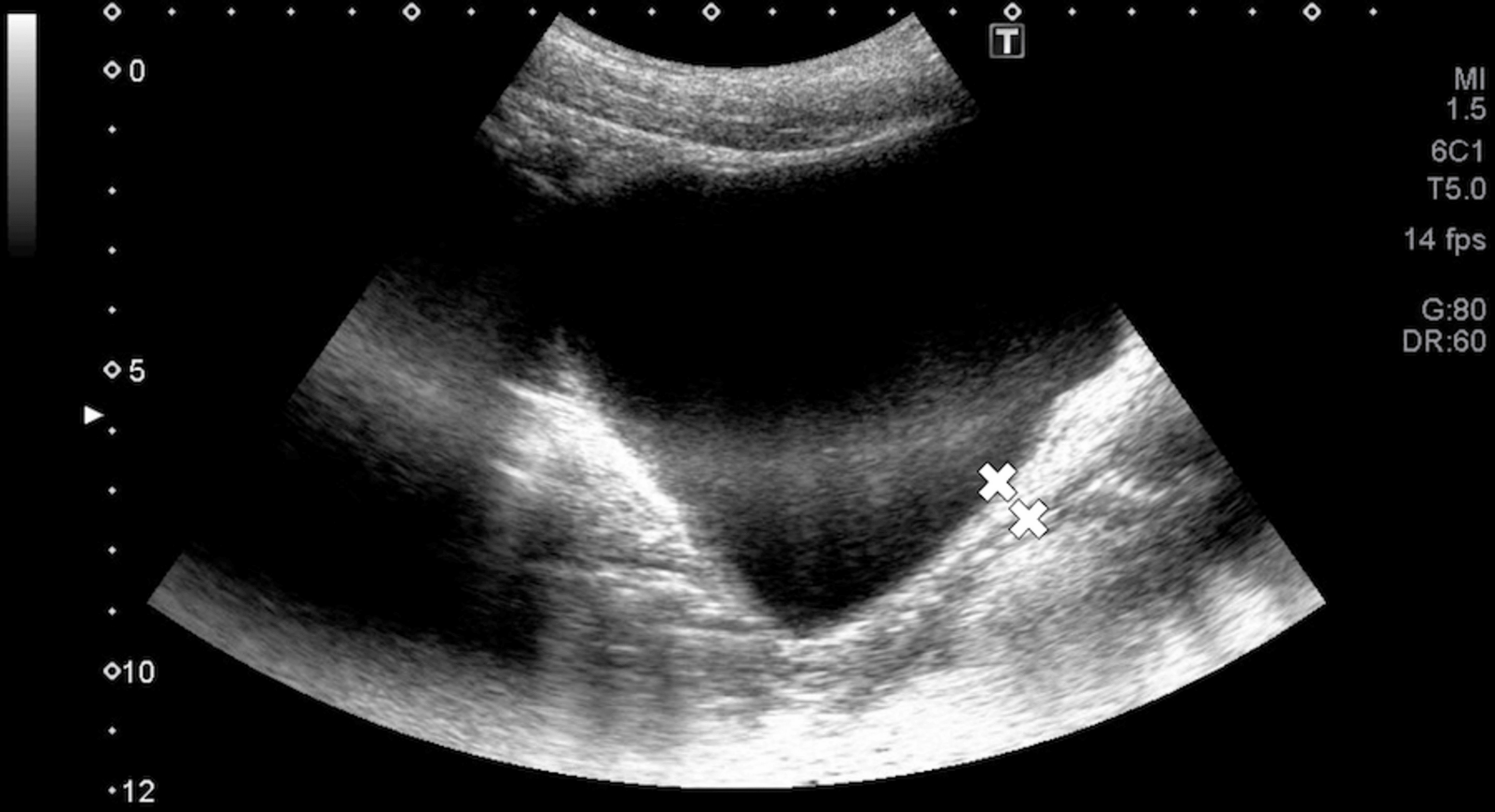
Ultrasound image of bladder base elevation during contraction (upper X mark indicates contraction, lower X mark indicates at rest) The point where the maximum displacement of the bladder base edge is clearly observed is designated as the reference point. At rest (a) and at maximum elevation (b), the reference point is indicated by “X.”

The degree of voluntary PFM contraction was assessed by measuring pelvic floor elevation using ultrasound, specifically the XARIO100 TUS-X100S (Toshiba Corporation, Minato, Tokyo, Japan) ultrasound system (convex electronic scan probe, B mode, frequency 3 Hz). To standardize bladder volume, participants were instructed to void prior to the measurement. The probe was positioned along the longitudinal axis and tilted at a 30° angle, 10 cm below the navel [21], and all procedures were conducted by a skilled female therapist. Throughout the measurement, additional female examiners ensured that the anterior-posterior pelvic tilt was in a neutral position. For privacy, the assessment area was enclosed by a curtain. To evaluate bladder base elevation, the clearly visible posterior bladder wall was identified, and the point of maximum displacement during the exercise - based on previous studies - was selected. This resting position was marked with an “X” (Figure 5). A tangent line was drawn from the “X” mark to the posterior bladder wall, and the perpendicular distance between the resting and elevated positions of the bladder was measured (Figure 6). Measurements were taken three times, and the average value was used for analysis [21].

Statistical analysis

A one-way analysis of variance was conducted to compare bladder base elevation across the four groups. When significant differences were identified, multiple comparisons were performed using the Bonferroni method. StatView (Version 5.0, SAS Institute, Cary, NC, USA) was used for statistical analysis. Normality was confirmed using the Shapiro-Wilk test, and a significance level of p < 0.05 was set for all comparisons.

Ethical considerations

This study was approved by the Research Ethics Review Committee of Kibi International University (21-30). The purpose of the study was thoroughly explained to the participants, and written consent was obtained before data collection. Given the sensitive nature of the measurement area, all explanations and measurements were conducted in collaboration with a female therapist to ensure privacy and comfort for the participants.

## Results

**Figure 7:**
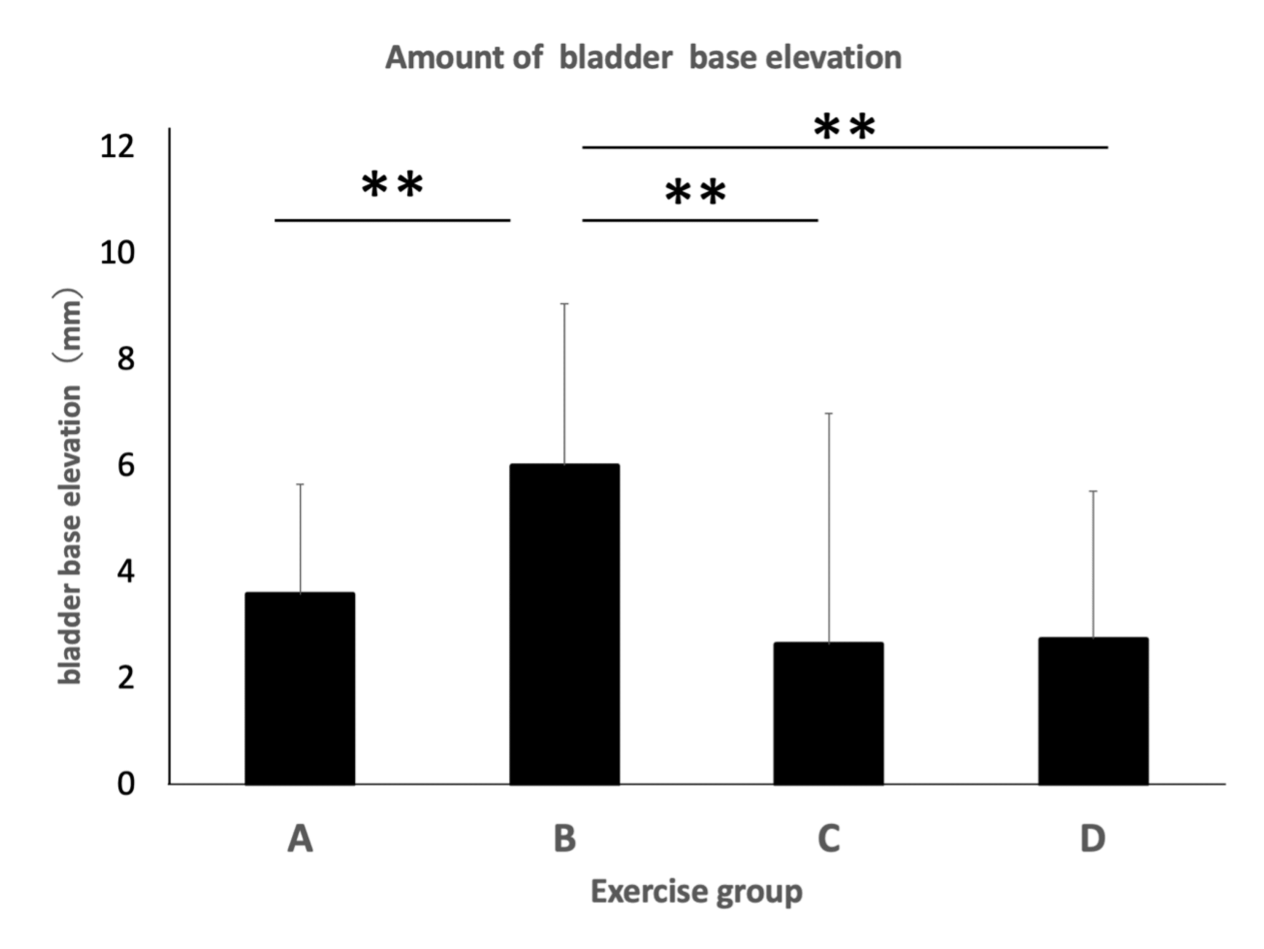
Bladder base elevation for each group ** p < 0.01 Means ± SD (n = 19 for each group) are shown.

The bladder base elevation was 3.58 ± 2.1 mm in Group A, 6.00 ± 3.1 mm in Group B, 2.64 ± 4.3 mm in Group C, and 2.73 ± 2.8 mm in Group D. Group B exhibited significantly higher bladder base elevation compared to all other groups (vs. A: p = 0.001, d = 1.46; vs. C: p = 0.001, d = 1.56; vs. D: p = 0.001, d = 1.61) (Figure 7).

In the individual PFM contraction task, Group B (supine kneeling position) showed greater pelvic floor elevation compared to Group A (supine position). However, in Group B, where pelvic floor contraction was followed by the supine kneeling position, and in Groups C and D, where hip abduction and hip adduction tasks were added, pelvic floor elevation was lower in Groups C and D than in Group B, where only the pelvic floor contraction task was performed.

## Discussion

In this study, we observed that Group B showed a significant increase in bladder base elevation compared to the other groups.

The increase in bladder base elevation in Group B, compared to Group A, is thought to be due to the tension induced in the internal obturator muscles and the tendon arch of the puborectalis muscle as a result of the hip flexion position. This likely made it easier for participants to be aware of the contraction of the anatomically connected puborectalis muscle. The puborectalis muscle, a part of the PFMs, is positioned deep within the pelvic floor and functions to lift the bladder base upon contraction.

Groups C and D, which involved co-contraction of the hip muscles, exhibited a similar decrease in lifting capacity despite assuming the hip flexion position, similar to Group B. This decrease can be attributed to the dual-task nature of simultaneously contracting both the PFMs and the hip muscles. When two tasks like these are executed concurrently and the combined demand exceeds capacity, it is suggested that the performance of one or both tasks may diminish [22]. This phenomenon, where the performance in one or both tasks constituting the dual task declines, is referred to as dual-task interference [23,24].

In dual-task situations, exercise performance is compromised because attention is divided. For example, studies on walking in the elderly have shown that simultaneously performing cognitive tasks, such as counting numbers in reverse order while walking, can slow walking speed and impair balance [25,26]. Dual tasks have also been reported to negatively affect skill accuracy and reaction time in athletes [27-29].

In this study, we believe that the dual task of co-contracting the challenging pelvic floor and hip muscles led to a decrease in bladder base elevation. Given the reduction in voluntary contraction of the PFMs due to this dual task, using low-frequency stimulation to activate the PFMs may be effective [11,30]. By contracting the PFMs in an altruistic manner with low frequency, it may become easier for participants to become aware of them and perform voluntary contractions. This remains an issue for future investigation.

The results of this study suggest that voluntary contraction of the PFMs may be more efficient in the upright kneeling position than in the supine position. Moreover, the contraction task of the PFMs alone, without joint contraction of the peri-hip muscles, may lead to more efficient voluntary contraction of the PFMs.

The limitations of this study include the fact that the subjects were young, healthy women, not patients with SUI or those from high-risk groups. It cannot be ruled out that co-contraction of the PFMs and hip adduction or abduction may facilitate voluntary contraction of the PFMs in patients with SUI or high-risk individuals. Future studies will focus on patients with SUI to verify differences in the effectiveness of PFM exercises that facilitate PFM contraction and their preventive effects on SUI.

## Conclusions

This study demonstrates that the efficiency of voluntary PFM contraction is significantly enhanced when exercises are performed in an upright kneeling position compared to a supine position. Specifically, the upright kneeling position alone, without co-contraction of the peri-hip muscles, resulted in the greatest bladder base elevation, suggesting that this posture optimally engages the PFMs. Conversely, simultaneous co-contraction of the hip adductors or abductors with PFM exercises led to reduced contraction efficiency, likely due to dual-task interference. These findings highlight the importance of considering limb positioning and muscle engagement strategies when designing effective PFM exercise protocols.

Future research should focus on validating these results in clinical populations, particularly among patients with SUI, to assess the generalizability and clinical applicability of these findings. Additionally, exploring the potential role of low-frequency stimulation as a supplementary technique for enhancing PFM awareness and voluntary contraction could open new therapeutic avenues.
